# Construction of Development Momentum Index of Financial Technology by Principal Component Analysis in the Era of Digital Economy

**DOI:** 10.1155/2022/2244960

**Published:** 2022-06-28

**Authors:** Boran Li, Xiao Gui, Qianyi Zhou

**Affiliations:** ^1^School of Public Policy and Management, Nanchang University, Nanchang 330000, China; ^2^School of Mathematics and Statistics, Changchun University of Technology, Changchun 130000, China

## Abstract

The purpose is to study applying mathematical analysis in financial technology (FinTech) development in the era of digital economy. An Evaluation Index System (EIS) for the current situation of Chinese FinTech enterprises is established by considering the impact of the era of the digital economy on the development of FinTech. Specifically, the Principal Component Analysis (PCA) is introduced to construct the principal component prediction model based on functional data. Then, six Chinese State-owned Enterprises (SOEs) are selected. Their stock prices are predicted using the proposed model through an empirical study. The results show that selecting three principal components to evaluate the financial situations of six SOEs is reasonable. The accumulated variance values of the first three principal components of the stock's closing price and opening price are all greater than 85%. Thus, the selected three principal components can obtain the potential information of the original data. The gap between the actual value and the proposed model-predicted value of the stocks of the six SOEs is relatively small. The Root Mean Square Error (RMSE) of China National Petroleum Corporation (CNPC) is 0.105, more than 10%. The predicted values of Huadian Energy and China Shenhua are 9.4% and 8.5%, respectively, second only to CNPC. Therefore, the proposed principal component prediction model based on functional data can predict the closing price of stocks well. The accuracy is relatively high and matches well with financial data analysis. This research has important implications for the development of FinTech.

## 1. Introduction

The era of digital economy has brought finance and technology ever closer. For example, digital technology services are involved in the financial growth model more effectively and integrated into the asset generation process of enterprises and individuals earlier. Under the digital economy, financial institutions' asset perspective ability and asset pricing ability can be fundamentally improved, helping financial institutions and real departments reduce costs, improve efficiency, enhance user experience, and optimize business models. Mathematical models have been widely used in the financial industry, and the academic branch of financial mathematics has been derived. Digital platforms are an enterprise organization model with natural monopoly tendencies. Internet giants often lack competition after gaining a monopoly position, which leads to the common problem of value defects in the platform economy [1–3]. The digital economy is a relatively broad concept. Its connotation changes continuously with the innovation of digital technology and people's understanding of the development of the digital economy [4–6]. All new industries, new formats, and new models with big data as the main feature generated by applying a new generation of information technology in economic activities can be included in the scope of the digital economy [7–9]. At the technical level, digital technologies such as big data, cloud computing, Internet of Things (IoT), blockchain, Artificial Intelligence (AI), and 5G communications have already penetrated all aspects of the economy and society. At the application level, the integration of the digital economy with various industries has accelerated, and new applications such as “new retail” and “new manufacturing” have been launched. The digital economy has realized the industrialization and marketization of the information technology revolution. It is more digital, networked, and intelligent than the traditional economy. In the era of the digital economy, data analysis is a basic tool in the financial industry. Accounting statements, bank flow, industry growth data, and industry risk samples must be analyzed in financial risk control and prediction [10–12]. It has great research value and significance.

Candraningrat et al. [13] pointed out that Small and Medium-sized Enterprises (SMEs) played a crucial role in promoting regional and national economic growth. Various types of SMEs were scattered across Indonesia, and the main issue was capital. The rapid growth of the financing business of financial technology (FinTech) was currently an alternative that is available to all levels of society through inclusive finance. This was a way of socializing the financial sector, especially by providing financial access to the public. Abad-Segura et al. [7] identified seven research directions in the study. They aimed to analyze aspects of finance, economics, technology transfer, investment, innovation, partnerships, institutions, and business. Research areas were sustainability analysis in banking, financial services trade, territorial development, law, management, research methods, and FinTech. As a core component of the digital economy, digital FinTech and ecological efficiency have a complex interaction and interdependence. Su et al. [14] analyzed the spatial interaction spillover effects of 284 Chinese cities from 2008 to 2018. They used simultaneous spatial equations and the three-stage ordinary least squares of generalized space from the perspective of overall spatial interaction. The results showed that digital FinTech and urban ecological efficiency promoted each other, and the latter was relatively dominant. Lv et al. [15] proposed the reliability of the Internet and the IoT and analyzed the control model based on the spatiotemporal correlation detection model. Lv and Qiao [16] analyzed the performance of a cooperative control system based on cognitive computing technology. Mora-Vega et al. [17] studied the ecosystem of the digital economy and e-commerce in Uruguay, especially related to the emergence of e-commerce and FinTech Companies. The research used secondary analytical data sources for qualitative and quantitative variables, promoting the potential of economic and financial formalization and inclusiveness. The research had important reference value for describing the financial technology ecosystem. Okfalisa et al. [18] summarized the digital preparation of SMEs and maintained their sustainable development by employing financial technologies. The results proved that the importance of financial transactions in the digital preparation of SMEs had contributed considerably to financial and technological applications in sustainable enterprise development. To sum up, the existing research has achieved some research results in the financial field. In particular, this work complements the existing research in FinTech and puts forward some suggestions for problems in the practical development of FinTech enterprises. The aim is to help FinTech enterprises develop better and faster.

Literature research and survey methods are adopted. By studying the impact of the arrival of the era of the digital economy on the development of FinTech, an Evaluation Index System (EIS) for the status quo of Chinese FinTech enterprises is established. The innovation lies in using mathematical analysis in financial data analysis and using the principal component prediction model based on functional data to predict and analyze stock prices, which has been verified to perform well.

The research framework consists of four parts. The first is the introduction, which introduces the current situation and research significance of the development of FinTech in the era of the digital economy. The research method part follows the introduction. By studying the impact of the era of the digital economy on the development of FinTech, an EIS for the current situation of Chinese FinTech enterprises is established. The Principal Component Analysis (PCA) is used to conduct a corresponding analysis. Thirdly, the results part counts and analyzes the data results of the PCA. Finally, the research is summarized, and the shortcomings of the research and future prospects are pointed out.

## 2. Materials and Methods

### 2.1. The Development of FinTech in the Era of the Digital Economy

**Figure 1:**
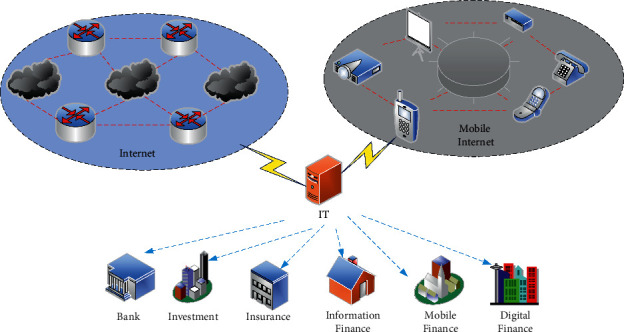
Financial composition in the era of the digital economy.

**Figure 2:**
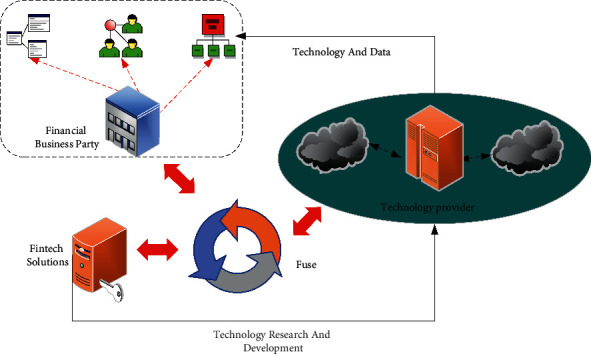
The relationship between FinTech development and enterprises.

The digital economy is the sum of a series of economic activities with digital technology as important content. It includes new industries, new formats, and new models spawned by the development of digital technology and new economic growth brought about by the deep integration and innovation of digital elements and traditional industries [19]. The core of the digital economy includes both digital technology and economic activities. A new generation of digital technologies has gradually formed a basic system that supports social and economic operations, including big data, cloud computing, IoT, blockchain, AI, and 5G. Essentially, the digital economy is an economic activity based on the development, utilization, and protection of data. It can assist or even replace human resources in a relatively de-intermediary, de-centralized, and trust-free environment in a more powerful way. The digital economy is not simply a “virtual economy” or a “future economy” independent of other economic systems or even opposed. Instead, it is a more advanced form of economic development based on the traditional economic system and promoted through technology as the core driving force. The financial structure in the era of the digital economy is shown in Figure 1:

The rapid development of China's digital economy is not only due to the development and innovation of new-generation digital technologies, including big data, cloud computing, IoT, blockchain, AI, and 5G communications but also to governments at all levels from the central to local governments actively promoting the development of the digital economy, providing extensive policy support for the construction of the digital economy. State-owned Enterprises (SOEs) take the lead in digital reform and play an exemplary role. The development of the digital economy is inseparable from financial support. In the digitization process, digital technology transforms the entire process of financial instruments, such as access/issuance standards, information disclosure, transactions, risk control, and other links. It deepens the understanding of the digital economy and improves financial efficiency. Meanwhile, financial institutions can reduce labor costs and subjective errors, improve accuracy and safety, and achieve digital traceability of the entire process by digitizing operations management. It has become a practitioner and beneficiary of the digital economy to serve the digital economy better. It denotes that finance in the era of the digital economy should be more inclusive, more digital, and more humane. All these require cooperation between business and government. The relationship between FinTech development and enterprises is shown in Figure 2:

Technological finance is a general term for various financial instruments promoting technological innovation and development. The development of technological finance is a major measure to implement the innovation-driven development strategy, build an innovative country, transform the mode of economic development, and adjust the economic structure. China's technology finance continues to improve, but technology and finance still need to be further integrated and developed. The regional development of technological finance shows that the technological finance system in first-tier cities is relatively sound, and the head effect is obvious. The rise of technological finance in some central and western cities is characterized by using its own advantages to promote the integration of technology and finance. It is necessary to strengthen policy guidance, improve the policy system, and guide social capital into the field of science and technology. It also needs to improve the financing capacity of enterprises and strengthen the innovation of financial institutions and their science and technological services.

### 2.2. Construction and Influencing Factors of the Development Momentum Index of FinTech

FinTech uses technological means to help the development of financial business. Major banks have successively started constructing a new generation of FinTech. Thus, informatization has become a must for business development. FinTech is based on a series of technological innovations such as big data, cloud computing, AI, and blockchain. It is fully applied to six financial fields: payment and settlement, loan financing, wealth management, retail banking, insurance, and transaction settlement, which is the future mainstream trend in the financial industry. Financial regulatory authorities in various countries continue to strengthen regulatory policies for risk prevention and control by applying new technologies in the financial sector. The regulatory policy is refined from technology, business, and entities, and the responsibilities of all parties are clarified. In the supervision of the risks of new technologies, the regulatory policies on the security protection of financial data are the most representative. On the one hand, countries continue to strengthen the top-level design and legislation of their financial data security and promote the formulation of financial data security standards. A third-party data security service agency is established to strengthen third-party risk assessment and vulnerability detection of financial institutions' data security capabilities. On the other hand, financial data protection has also become the focus of international coordinated supervision.

In terms of FinTech investment, the growth rate of financial institutions' technology investment far exceeds the growth rate of their income. With the continuous deepening application, FinTech has gradually begun to drive the transformation of the organizational structure of financial institutions. Especially in the face of the new digital transformation and development situation, the transformation of organizational structure has become an inevitable trend.

As a new field of integration of finance and technology, many factors affect its development. In particular, the key to evaluating FinTech enterprises' development is to determine the evaluation indexes. Here, a comprehensive evaluation is carried out combined with the overall operation status of China's financial industry and the development capabilities of enterprises. Consequently, an EIS is established to target financial development's quality, efficiency, and potential. Combined with the diversity of Chinese financial enterprises, the established EIS considers four aspects: operating capability, capital operating capability, profitability, and Research and Development (R&D) capability, which can reflect the operating ability and economic level of the enterprise to a certain extent.  Table 1 shows the specific evaluation indexes of the four criteria:

In Table 1, 4 first-level indexes and 12 second-level indexes have been established to evaluate the operation and management status of enterprises. Among them, the growth rate of operating income represents the ratio of the growth of the enterprise's operating income in the current year to the total operating income of the previous year. It reflects the increase or decrease in the operating income of the enterprise. A growth rate greater than 0 indicates that the enterprise's operating income is increasing in the current year. The larger the growth rate is, the better the development of the enterprise is. The average three-year growth rate of operating income reflects the state and trend of the enterprise's sustainable development and the ability to expand the market. The larger the value is, the better the expansion trend of the enterprise is. The proportion of technology investment reflects the ratio of the enterprise's technology expenditure to operating income, including the R&D of new products, the transformation of existing technical equipment, or high-tech innovation and other fields. The higher the value is, the better the future development prospects of the enterprise is. A higher employment ratio of highly educated talents such as undergraduates, master's, and doctorates means the enterprise's overall strength is relatively high, and it can attract highly educated talents to join.

Assuming that the base year is 2010 and the full score of each index for that year is 100, the calculation methods of each index are as follows:

(1) $$\begin{array}{c} {\text{Score}}_{i}=\frac{X_{i}-X_{\min}}{X_{\max}-X_{\min}}*100. \end{array}$$

*X*_*i*_ represents the value of the *i*th index in a certain place. *X*_min_ refers to the minimum value of the *i*th index of each FinTech enterprise in the base year, and *X*_max_ indicates the maximum value. The nonbase year index can show the time trajectory of the FinTech development index status of FinTech enterprises. For the calculation of various indexes in the nonbase year, the score of the *i*th index in *t* year is calculated, as shown in the following equation:

(2) $$\begin{array}{c} {\text{Score}}_{i}^{t}=\frac{X_{i\left(t\right)}-X_{\min\left(t\right)}}{X_{\max\left(t\right)}-X_{\min\left(t\right)}}*100. \end{array}$$

In a rapidly growing and large-scale economy, stable economic and social development will provide a good environment for enterprises. Generally, the higher the liquidity of funds is in economically developed areas, the lower the probability of financial lending is. However, in underdeveloped areas, the development speed of FinTech, an industry that requires a lot of scientific research investment and financial support, will inevitably be limited. FinTech combines advanced Internet and communication technologies to obtain more transactions. However, the network bandwidth and network stability still need to be improved. In the face of more and more potential customers, technological innovation is required to meet the financial needs of users and occupy more market share.

### 2.3. Principle and Prediction Method of PCA

Principal Component Analysis (PCA) can be regarded as a kind of projection that maps the data in the high-dimensional space to the low-dimensional space. For a set of multivariate data, the internal structure of the data is revealed by changing the angle of data observation. This process is called PCA. PCA is a basic mathematical analysis method. Its practical application is extensive, including demographics, molecular dynamics, mathematical modeling, mathematical analysis, quantitative investment, and other disciplines. It is a commonly used multivariate analytical method. In multivariate data analysis, too many variables can greatly increase the complexity of the problem. In addition, there is a certain linear correlation between variables in many problems, which causes the overlapping of data information represented by different variables. Against these problems, the natural idea is to use fewer but uncorrelated variables to obtain as much information about the original data as possible. Starting from the original variables, PCA constructs a set of new, uncorrelated new variables through rotational changes, namely, the linear combination of the original variables. These variables explain the differences between the original data as much as possible, that is, the internal data structure; they are called principal components of the original data. Since these variables are uncorrelated, they explain some of the variability without overlapping. They are called the first principal component, the second principal component, and so on, ordered by the magnitude of the difference explained by each variable.

Kaiser–Meyer–Olkin (KMO) and Bartlett's tests are required before performing PCA on the original data. Usually, SPSS software is used to conduct factor analysis. If the value of KMO is greater than 0.5, factor analysis can be performed. If it is greater than 0.7, it is more in line with the standard of factor analysis. The specific calculation method for PCA is first to assume that the original sample data matrix is:

(3) $$\begin{array}{c} X=\left[\begin{array}{cccc} x_{11} & x_{12} & \ldots & x_{1p}\\ x_{21} & x_{22} & \ldots & x_{2p}\\ \ldots & \ldots & \ldots & \ldots \\ x_{m1} & x_{m2} & \ldots & x_{mp} \end{array}\right]. \end{array}$$

On this basis, the original sample data are normalized to eliminate the dimension of the data. The expression is shown in the following equation:

(4) $$\begin{array}{c} \left\{\begin{array}{c} x_{ij}^{*}=\frac{x_{ij}-\bar{x_{j}}}{\sqrt{\text{Var}\left(x_{j}\right)}},\\ i=1,2,\ldots ,m,\\ j=1,2,\ldots ,p. \end{array}\right. \end{array}$$

The calculation method is shown in equations (5) and (6):

(5) $$\begin{array}{c} {\bar{x}}_{j}=\frac{1}{m}\sum_{i=1}^{m}x_{ij}, \end{array}$$

(6) $$\begin{array}{c} \left\{\begin{array}{c} \text{Var}\left(x_{j}\right)=\frac{1}{m-1}\sum_{i=1}^{m}{\left(x_{ij}-{\bar{x}}_{j}\right)}^{2}\\ j=1,2,\ldots ,p. \end{array}\right. \end{array}$$

Furthermore, it is necessary to calculate the correlation coefficient matrix of the sample, and the calculation method is as follows:

(7) $$\begin{array}{c} R=\left[\begin{array}{cccc} r_{11} & r_{12} & \ldots & r_{1p}\\ r_{21} & r_{22} & \ldots & r_{2p}\\ \ldots & \ldots & \ldots & \ldots \\ r_{m1} & r_{m2} & \ldots & r_{mp} \end{array}\right],\\ \left\{\begin{array}{c} r_{ij}=\frac{1}{m-1}\sum_{t=1}^{m}X_{ti}X_{tj},\\ i,j=1,2,\ldots ,p. \end{array}\right. \end{array}$$

Then, the eigenvalues and eigenvectors of the correlation coefficient matrix *R* are solved to obtain the result:

(8) $$\begin{array}{c} \left\{\begin{array}{c} a_{i}=\left(a_{i1},a_{i2},\ldots ,a_{ip}\right),\\ i=1,2,\ldots ,m. \end{array}\right. \end{array}$$

The eigenvalue size represents the amount of information contained. The obtained eigenvector denotes the weight coefficient of the original index variable on the principal component. Then, the corresponding principal component factor is extracted. Next, the principal component factors are interpreted according to their contribution rate, and the final calculation expression is obtained.

The general multivariate PCA structure is to linearly combine multiple variables and quickly perform the information extraction process from many variables. However, the variance-covariance and related functions obtained in this way cannot explain the variable structure in the observed data very well. The PCA method of functional data can provide more information. It can improve or even replace the analysis results of the variance-covariance function.

Assuming that in the PCA, the variable value function is *x*(*s*), the function matrix is {*x*_1_(*s*), *x*_2_(*s*),…, *x*_*N*_(*s*)}, and the discrete index *i* is used instead of the continuous index *s*. The score of the principal component is calculated as shown in the following equation:

(9) $$\begin{array}{c} f_{i}=\int \beta \left(s\right)x_{i}\left(s\right)\text{d}s. \end{array}$$

*β*(*s*) means the weight function, and *x*_*i*_(*s*) denotes the curve function. According to the dimension of the original data, the PCA is divided into one-variable and muti-variable functions. One-variable PCA first smoothens the original data, and the variance and covariance are also used to express the internal changes in the data. The variance calculation method is as follows:

(10) $$\begin{array}{c} \text{Var}\left(f\right)=\frac{1}{n}\sum_{i=1}^{n}\left({\int \beta x_{i}}^{2}\right). \end{array}$$

*n* refers to the dimension of the data matrix. To find the first principal component *f*_*i*_, it needs to meet equations (11) and (12):

(11) $$\begin{array}{c} \text{max}:\text{Var}\left(f_{1}\right)=\frac{1}{n}\sum_{i=1}^{n}\left({\int \beta \left(s\right)x_{i}\left(s\right)\text{d}s}^{2}\right), \end{array}$$

(12) $$\begin{array}{c} s.t.{\left\|\beta \right\|}^{2}=\int \beta \left(s\right)\beta \left(s\right)\text{d}s=1. \end{array}$$

In this way, the weight function of the first principal component *β*^2^(*s*) is obtained, and the *k*th principal components *f*_*k*_ can be obtained in the same way. The weight function is solved by using the sample covariance matrix function to find the weight function of the principal components. The calculation method of the covariance function is shown in the following equation :

(13) $$\begin{array}{c} v\left(s,t\right)=\frac{1}{n}\sum_{i}x_{i}\left(s\right)x_{i}\left(t\right). \end{array}$$

Then, the characteristic equation of the weight function is shown in the following equation:

(14) $$\begin{array}{c} \int v\left(s,t\right)\beta \left(t\right)dt=\lambda_{i}\beta \left(s\right). \end{array}$$

*λ* shows the eigenvalue of the covariance function *v*(*s*, *t*). In the PCA, the number of principal components is determined according to the cumulative contribution rate, which usually requires a cumulative contribution rate of more than 85%. The calculation method of the cumulative contribution rate of the *K*th principal component is shown in the following equation:

(15) $$\begin{array}{c} \frac{\sum_{i=1}^{K}\lambda_{k}}{tr\left(V\right)}. \end{array}$$

The PCA method of the function of several variables is extended based on the willingness function. In the binary function, for example, the variables are represented by *x*(*s*), *y*(*s*). The linear integral of the principal components of the two variables is shown in the following equation:

(16) $$\begin{array}{c} f_{i}=\int \beta_{1}\left(s\right)x_{i}\left(s\right)ds+\int \beta_{2}\left(s\right)y_{i}\left(s\right)ds. \end{array}$$

*β*_1_(*s*), *β*_2_(*s*) means the covariance of *x*(*s*), *y*(*s*). Actual financial data are usually random, so using the functional PCA model for prediction is more convenient.

Assuming that the stochastic process {*X*(*t*) : *t* ∈ [*T*_1_, *T*_2_]} has continuity and can be integrated multiple times, for a given variable {*X*(*t*) : *t* ∈ [*T*_1_, *T*_2_]}, it is necessary to obtain a linear estimate of the random variable, and build a model for its changing trend in the interval [*T*_3_, *T*_4_]. The random variable $\overset{⟶}{X}\left(\text{s}\right)$ is the mean estimate of the random variable *X*(*s*), then *ε*^2^(*s*) is the RMSE generated by the linear estimation at the *s* ∈ [*T*_3_, *T*_4_] moment; the expression is shown in the following equation :

(17) $$\begin{array}{c} \varepsilon^{2}\left(\text{s}\right)=E{\left[\left|\overset{⟶}{X}\left(s\right)X\left(s\right)\right|\right]}^{2}. \end{array}$$

After the variable interval is selected, the respective principal components of the two intervals are calculated. The *i*-th principal component of the random variable *X*(*t*) in the interval *t* ∈ [*T*_1_, *T*_2_] is expressed as:

(18) $$\begin{array}{c} \xi_{i}=\int_{T_{1}}^{T_{2}}\left(X\left(t\right)-u\left(t\right)\right)f_{i}\left(t\right). \end{array}$$

*f*_*i*_ denotes the principal component factor and *u*(*t*) indicates the mean of *X*(*t*). V means the total variance of the variable in the interval [*T*_1_, *T*_2_], and the expression is shown in the following equation:

(19) $$\begin{array}{c} V=E\left[\int_{T_{1}}^{T_{2}}{\left(X\left(t\right)-u\left(t\right)\right)}^{2}\text{d}t\right]. \end{array}$$

The future time interval is denoted by [*T*_3_, *T*_4_], then the linear least squares estimate $\overset{⟶}{X}\left(\text{s}\right)$ of *X*(s) is shown in the following equation:

(20) $$\begin{array}{c} \overset{⟶}{X}\left(s\right)=u\left(s\right)+\sum_{j=1}^{\infty }\overset{⟶}{\eta_{j}}g_{i}\left(s\right). \end{array}$$

$\overset{⟶}{\eta_{j}}$ illustrates the linear representation of the random variable on the interval. *g*_*i*_ and *g*_*i*_ stand for the characteristic function and principal component on the corresponding interval. Then, the final principal component prediction model is shown in the following equation:

(21) $$\begin{array}{c} \overset{⟶}{X^{q}}\left(s\right)=u\left(s\right)+\sum_{j=1}^{\infty }\overset{⟶}{\eta_{j}^{p_{j}}}g_{i}\left(s\right). \end{array}$$

*p*_*j*_ represents the first few principal components, and *q* expresses the number of principal components. Finally, the model evaluation indexes select the percentage error, the Mean Absolute Percentage Error (MAPE), and the Root Mean Square Error (RMSE). The percentage error is represented by *u*_*j*_, and the expression is shown in the following equation:

(22) $$\begin{array}{c} u_{j}=\frac{X_{i}-{\overset{⟶}{X}}_{i}}{X_{i}}*100\%. \end{array}$$

The MAPE is expressed by *u*^*∗*^, and the expression is shown in the following equation:

(23) $$\begin{array}{c} u^{*}=\frac{1}{n}\sum_{i=1}^{n}\left|u_{j}\right|. \end{array}$$

*u*_*j*_ refers to the percentage error, and the expression of RMSE is shown in the following equation:

(24) $$\begin{array}{c} \text{RMSE}=\sqrt{\frac{\sum_{i=1}^{n}{\left(X_{i}-{\overset{⟶}{X}}_{i}\right)}^{2}}{n}}. \end{array}$$

The larger these three indexes are, the better the performance of the model is. In equations (22)–(24), *X*_*i*_ denotes the observed true value, ${\overset{⟶}{X}}_{i}$ means the predicted value obtained by the model, and *n* stands for the dimension of the data matrix.

### 2.4. Stock Prediction Based on the Lightweight Deep Learning Model

With science and technological advancement, diversified models can predict the fluctuation trend of stock prices. Its development process is mainly: the traditional time series model, machine learning model, and deep learning neural network model. In the early stock market, the prediction of the stock price trend is usually carried out using the traditional time series model. However, due to the high noise and nonlinear characteristics of time series, the accuracy of the stock price trend predicted by this model is not high. With the emergence of machine learning technology, many scholars apply machine learning models to predict stock prices and find that the prediction results are more accurate than those of traditional time series models. However, traditional machine learning algorithms need to implement complex and effective learning models, which will consume great work and time in the data preprocessing stage. To solve these problems, scholars began to introduce their research directly into the field of deep learning neural networks. They find that the derivative of the Recurrent Neural Network (RNN) model—the Long Short-Term Memory (LSTM) neural network model—has the best effect. Moreover, it can well solve the problems of gradient disappearance and inability to obtain the optimal solution, realizing the consideration between time series and nonlinearity of data. Therefore, it has certain advantages in processing the time series data of stocks and can well fit the fluctuation law of stock price. Nevertheless, the LSTM model's calculation cost is quite high, which cannot be tolerated in tasks with strict waiting time requirements. There are still some challenges in real-time and accurate stock forecasting. Because of the above limitations of the LSTM network, a new RNN model based on the Minimum Gated Unit (MGU) is proposed. MGU has the advantages of simple structure, fewer parameters, and less training time. It is especially suitable for tasks with strong time dependence. Meantime, using an MGU model with fewer parameters can reduce the workload of selecting parameters and improve the model's generalization ability.

The proposed algorithm flow is presented as follows:

  Step 1. preprocess and normalize the network traffic matrix data sampled from the dataset;
  Step 2. select a continuous dataset from the two preprocessed datasets, divide the selected part of the data into the training set and test set, and use the sliding window method mentioned in the model design to obtain the data input by the model each time;
  Step 3. initialize the weight of the MGU model randomly, train the model with the training dataset, and adjust the parameters in the model according to the model loss function until the model reaches the target accuracy;
  Step 4. use the test set to test the trained MGU model, and fine-tune the MGU model according to the test set's performance to obtain the ideal model.

### 2.5. Empirical Analysis of Stock Prices

From January to December 2021, the data of closing price and opening price of the stock of 6 SOEs, China National Petroleum Corporation (CNPC), SINOPEC Group, National Power, Huadian Energy, China Construction, and China Shenhua are used. There are 256 transactions, from which the first 200 data are selected to form the training set, and the last 56 data are used as the verification set. The training set of opening prices is marked with [*T*_1_, *T*_2_], the training set of closing prices is marked with [*T*_3_, *T*_4_], and PCA is performed on the collected data. The KMO value of the collected data is 0.835 > 0.7, which meets the requirements and can be used for factor analysis. The six stocks are profitable as a whole, and the stock price will be partially disturbed during holidays. After the holidays, it will gradually stabilize.

## 3. Results and Discussion

### 3.1. Comparison of Research Data on the Stocks of 6 SOEs

**Figure 3:**
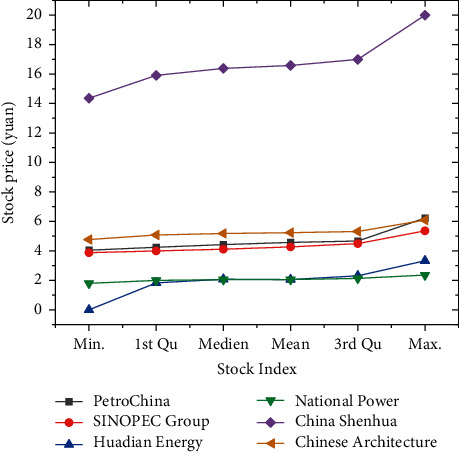
The characteristic index of the closing prices.

The characteristic index of the closing prices of the six SOEs is shown in Figure 3:

In Figure 3, 1st Qu represents the 1/4 digit, which is the upper quartile, and 3rd Qu refers to the 3/4 digit, which is the lower quartile. The comparison indicates that the stock price of China Shenhua is the highest among the six enterprises, with the highest price of 19.990, the lowest price of 14.360, and the average stock price of 16.580. Shares in several other enterprises are largely flat.

**Figure 4:**
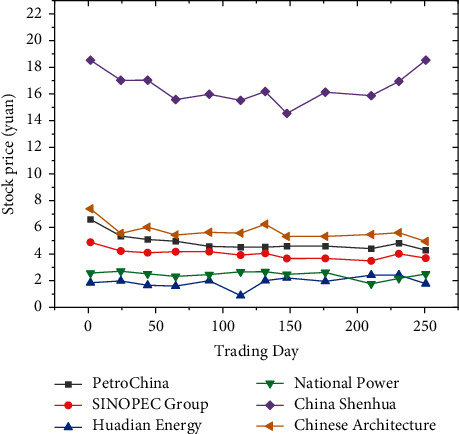
Point plot of closing prices of 6 SOEs.

**Figure 5:**
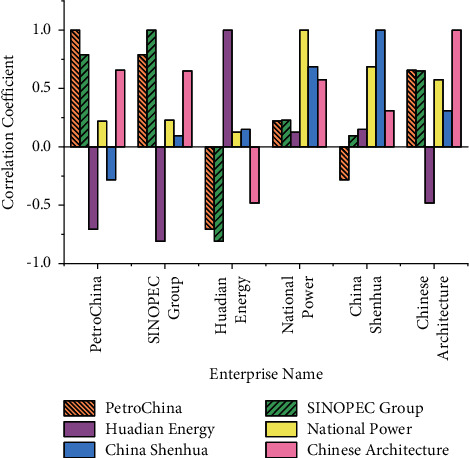
The correlation between the various stocks.

Before the study, the stock data need to be preprocessed, and the basis function smoothing method is used. The original closing price data is shown in Figure 4:

Figure 4 demonstrates that except for China Shenhua, as time changes, the stock prices of several other companies show a relatively stable fluctuation range. Therefore, the basis function is selected to expand the B-spline basis function. The correlation between the various stocks is shown in Figure 5:

In Figure 5, the companies with higher correlation are CNPC and SINOPEC Group. The correlation coefficient between them is 0.790, and the correlation coefficient between China Shenhua and National Power is 0.687. In contrast, the stock correlation coefficient between Huadian Energy and SINOPEC Group is negatively correlated, with a value of −0.811. The correlation coefficient between SINOPEC Group and Huadian Energy is also negative, with a value of −0.811. The correlation coefficient between China Railway and China Shenhua is also negatively correlated, with a value of −0.180. Thus, to reduce the probability of overlapping information, the dimensionality reduction of PCA is used for analysis.

### 3.2. PCA of Stock Price Based on Fitting Function

Because the closing price and opening price of the stock are continuous, the opening price training set [*T*_1_, *T*_2_] and the closing price training set [*T*_3_, *T*_4_] of 200 data in the sample training set are used for PCA. It is reasonable to choose three principal components here. From the principal component disturbance map made by the statistical software, it can be found that the variance contribution rate of the first principal component is 62.3%. The variance contribution rates of the second principal component and the third principal component are 14.7% and 11.2%, respectively. The sum is 88.2%, which can include most of the data information after fitting, reflecting the reasonable selection of principal components.

**Figure 6:**
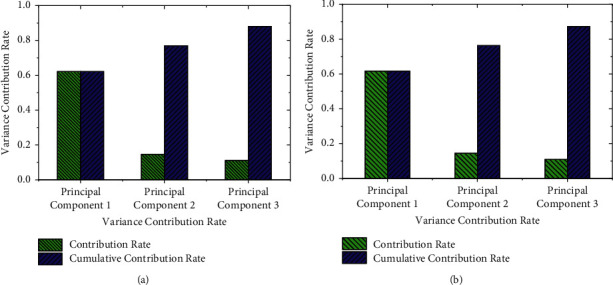
The variance contribution rate of principal component ((a)[*T*_3_, *T*_4_]; (b)[*T*_1_, *T*_2_]).

**Figure 7:**
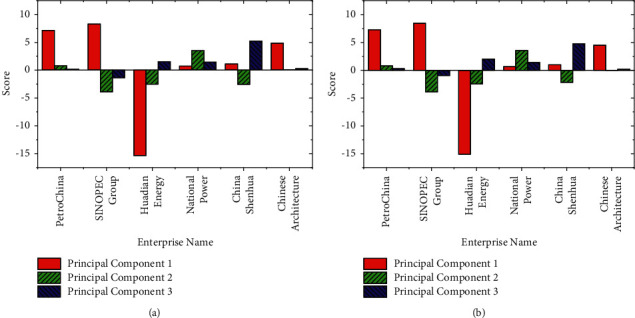
The score of the principal component ((a)[*T*_3_, *T*_4_]; (b)[*T*_1_, *T*_2_]).

**Figure 8:**
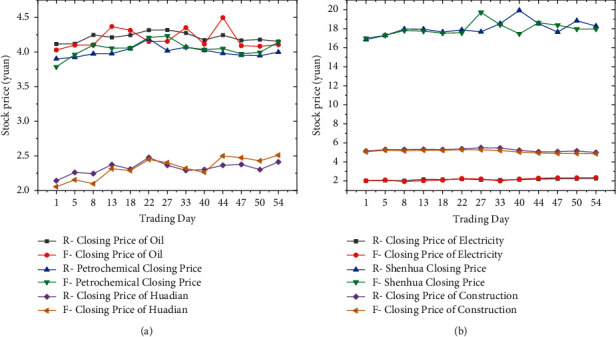
Comparison of forecast value and real value closing prices of 6 SOEs.

**Figure 9:**
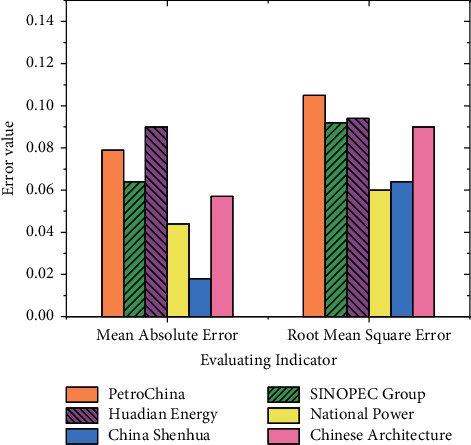
The MAPE and RMSE of the forecast closing price of 6 SOEs.

The variance contribution rate of the principal component and score of the opening price and closing price is obtained by using the function-based principal component prediction model, as shown in Figures 6 and 7:

Figures 6 and 7 imply that the cumulative variances of the first three principal components of the stock closing price and opening price are all greater than 0.850. Thus, these three principal components can obtain the potential data information of the original data. The closing price after 56 days is predicted by the prediction model, as shown in Figure 8:

Figure 8 manifests that there is a small gap between the actual closing price of the stocks of these six SOEs and the predicted value made by the model. Among them, the deviation between the predicted value and the actual value of CNPC and SINOPEC Group is relatively large. However, the overall trend is relatively stable, indicating that the prediction accuracy of the established closing prediction model according to the opening price is relatively high. The MAPE and RMSE of the forecast closing price of each enterprise are calculated for intuitive demonstration, as shown in Figure 9:

Figure 9 demonstrates that the RMSE of CNPC is 0.105, which is above 0.010. It is relatively high. The RMSE of the other five enterprises is below 0.010. Among them, the predicted values of Huadian Energy and China Shenhua are 0.094 and 0.085, respectively, second only to CNPC. It means that the principal component prediction model based on functional data can predict the closing price of stocks well. The accuracy is relatively high, which further shows that the prediction model matches financial data analysis well.

## 4. Conclusion

Digitization and informatization are the most prominent characteristics of the digital economy. The digital economy also attributes technology's natural democracy and innovation. The advancing state-of-the-art technologies such as blockchain and AI have quickly entered the financial field. This work studies the impact of the era of the digital economy on the development of FinTech. As a result, an EIS for the current situation of Chinese FinTech enterprises is established along with a calculation method for the development index. Then, a PCA is carried out on the stock prices of six SOEs in China. Finally, the principal component prediction model based on functional data is built to predict the stock price trend. The results manifest that the established model has good prediction performance. The RMSE of CNPC is 0.105, which is above 10% and is relatively high. The RMSE of the other five enterprises is below 10%. Among them, the predicted values of Huadian Energy and China Shenhua are 9.4% and 8.5%, respectively, second only to CNPC. It means that the principal component prediction model based on functional data can predict the closing price of stocks well, and the accuracy is relatively high.

Under a virtuous cycle, all participants in the financial system can benefit from it. The entire financial industry will develop healthily, which is also an inexhaustible driving force for the development of FinTech. However, there are still some shortcomings. As an emerging industry in the era of the digital economy, the history of the Internet finance industry is not long, but it has a strong development momentum and broad development space. In recent years, credit risk problems have also gradually increased. China should strengthen the credit risk measurement of the industry and detect credit risks early. Problems such as defaults that may be caused by credit risk should be solved in infancy. In addition, although the functional data analysis method has its advantages, it belongs to a relatively new field. More examples are needed to verify the mastery of this method in the future.

## Figures and Tables

**Table 1:** EIS of the development status of FinTech enterprises.

Basic criteria	Indexes	Coding of indexes	Basic criteria	Indexes	Coding of indexes
Operating capability	The growth rate of enterprise income	I1	Capital operating capability	The growth rate of capital preservation	I7
	Average three-year growth rate of earnings	I2		Accumulation rate of capital	I8
	The growth rate of total assets	I3		Average three-year growth rate of capital	I9

Profitability	Profit growth rate	I4	R&D capability	The proportion of technology investment	I10
	Return on equity	I5		The proportion of employees with a bachelor's degree or above	I11
	Earnings per share	I6		The proportion of master's and doctorates among executives	I12

## Data Availability

All data are fully available without restriction.
